# Inappropriate Journal Authorship

**DOI:** 10.5041/RMMJ.10513

**Published:** 2023-10-29

**Authors:** Marshall A. Lichtman

**Affiliations:** University of Rochester Medical Center, James P. Wilmot Cancer Institute, Rochester, New York, USA

**Keywords:** Authorship disputes, inappropriate journal authorship, institutional trust

**To the Editor:**

In their article “Authorship Disputes in Scholarly Biomedical Publications and Trust in the Research Institution” in the July 2023 issue of *Rambam Maimonides Medical Journal*, Ashkenazi and Olsha examined the association between the prevalence of misattributed authorship and trust in the institution analyzing misconduct in their scholarly publications.^1^ The authors appropriately include “gift authorship” as one of the three principal deviations from appropriate authorship choices that they examined. In essence, gift or honorary authorship is listing an author on a scholarly publication for which that person’s contribution did not justify assigning authorship. This behavior has become commonplace.^2^,^3^

For a time, I collected two types of articles displaying exaggerated authorship: (1) papers in which the number of authors exceeded the number of patients studied, usually in a small clinical trial; and (2) reports of single cases in which the authorship consisted of ten or more authors.^4^

The ingenuity and creativity of justifying authorship in the cases in which the journal required such an explanation impressed me. In recognition of those skills, I have offered the Marshall A. Lichtman Prize to the senior author.^5^ The latest prize went to the lead author of an article that listed 22 individuals as “authors” in the description of the treatment of a single case.^5^ The previous prize was awarded to the senior author who listed 20 authors of the report of a single case.^5^,^6^ The latter lead author creatively included the (lab) administrator as an author. There is no monetary component to this prize. It is a small statue of Diogenes of Sinope, the Greek philosopher, carrying a lantern as he searches for an honest person.

Initially Kovacs, and then I, have provided, independently, a solution to the disturbing trend of inappropriate “gift” or “honorary” authorship.^6^,^7^ Our suggestion is to assign to the author the fraction of a paper based on the number of authors. If, for example, there are four authors, each gets one-quarter of a paper, unless they assign different proportions to themselves, but totaling one paper. If, for example, the authors agree that the first and last, or fourth, author should be assigned 0.4 and 0.4 fraction of the paper based on their relative contribution, the second and third authors would be assigned a 0.1 fraction of a paper each, all together totaling one paper. This approach would provide a compelling incentive to include only authors of substance and not colleagues who may deserve acknowledgement. So, for example, the authors of a paper might be cited as Jones AB (0.5), Smith CD (0.25), and Brown FA (0.25), delineating their fractional contributions to the paper, but totaling one paper. The application of such a system would allow the authors, themselves, to assign the quantitative nature of their contribution. These data would provide the author’s citation record; they are explicit and numerical. Derivative indices can be based on either citations or high-impact citations. This approach should enhance the meaningfulness of authorship. It may take time to accumulate and replace the data on citations that exist in the current (flawed) system, but it would be a more meaningful mechanism to delineate authorship. It would ensure the best possible meaning to the designation “author.”

The law of conservation of mass requires the mass of the products to be equal to the mass of the reactants. Under our suggested new system, the number of citations would not exceed the number of papers. It is inappropriate, indeed misleading, to generate multiple papers from one paper, as the current citation process does; and, it fosters the practice of adding undeserving authors. There is no cost to the appropriate authors to add any number of undeserving authors in an act of collegiality or a group effort to inflate citations,^2^ representing a failure to understand their ethical obligation. There is, also, sometimes, the (unethical) requirement (pressure) to include a senior person who was not a meaningful contributor.^3^

Kovacs has provided the most in-depth discussion of the pros and cons of the proposal to have the citation credit of all authors on a paper sum to one paper.
